# Asociaciones público-privadas en el sistema de salud colombiano: dispositivos institucionales y disputas por la gestión de la salud

**DOI:** 10.1590/0102-311XES014125

**Published:** 2026-08-03

**Authors:** Mónica Uribe-Gómez, Yadira Borrero-Ramírez, Adriana Mendoza-Ruíz

**Affiliations:** 1 Facultad de Ciencias Humanas y Económicas, Universidad Nacional de Colombia, Medellín, Colombia.; 2 Facultad Nacional de Salud Pública, Universidad de Antioquia, Medellín, Colombia.; 3 Escola Nacional de Saúde Pública Sergio Arouca, Fundação Oswaldo Cruz, Fiocruz, Rio de Janeiro, Brasil.

**Keywords:** Sistemas de Salud, Asociación entre el Sector Público-Privado, Política Sanitária, Gestión en Salud, América Latina, Health Systems, Public-Private Sector Partnerships, Health Policy, Health Management, Latin America, Sistemas de Saúde, Parcerias Público-Privadas, Política Sanitária, Gestão em Saúde, América Latina

## Abstract

Este artículo indaga por la expansión de las alianzas público-privadas en el sistema de salud colombiano, enfocándose en los dispositivos institucionales de las políticas sanitarias que facilitan la participación del sector privado en la prestación de servicios y la gestión de recursos. Se analizan las reformas legales y políticas que han impulsado estas alianzas, así como las disputas entre actores públicos y privados por el enfoque y funcionamiento del sistema de salud. El estudio busca entender cómo este tipo de alianzas ha impactado la gestión de la salud, el acceso y la provisión de los servicios. Se realizó un estudio de caso con análisis temático de documentos oficiales, sentencias de la Corte Constitucional y notas de prensa, complementados con una revisión crítica de la literatura especializada. Los hallazgos indican que los mecanismos institucionales han favorecido la consolidación del sector privado, facilitando su acceso a recursos financieros y la formación de redes estratégicas para legitimar la perspectiva empresarial en la salud.

## Abstract (en)

This article explores the expansion of public-private partnerships in the Colombian healthcare system, focusing on the institutional mechanisms of healthcare policies that facilitate private sector participation in service provision and resource management. The study analyzes the legal and political reforms that have driven these partnerships, as well as the disputes between public and private actors over the approach and functioning of the healthcare system. The study seeks to understand how these types of alliances have impacted health management, access, and service provision. A case study was conducted with a thematic analysis of official documents, Constitutional Court rulings, and press releases, complemented by a critical review of the specialized literature. The findings indicate that institutional mechanisms have favored the consolidation of the private sector, facilitating its access to financial resources and the formation of strategic networks to legitimize the business perspective in health.

## Resumo

Este artigo investiga a expansão das parcerias público-privadas no sistema de saúde colombiano, com foco nos dispositivos institucionais das políticas de saúde que facilitam a participação do setor privado na prestação de serviços e na gestão de recursos. São analisadas as reformas legais e políticas que impulsionaram essas parcerias, bem como as disputas entre atores públicos e privados em relação ao enfoque e ao funcionamento do sistema de saúde. A pesquisa busca compreender como esse tipo de aliança tem impactado a gestão da saúde, o acesso e a prestação de serviços. Foi realizado um estudo de caso com análise temática de documentos oficiais, decisões do Tribunal Constitucional e notas de imprensa, complementado por uma revisão crítica da literatura especializada. Os resultados indicam que os mecanismos institucionais favoreceram a consolidação do setor privado, facilitando seu acesso a recursos financeiros e a formação de redes estratégicas para legitimar a perspectiva empresarial na saúde.

## Introducción

Las asociaciones público-privadas (APP) se consolidaron como modelo de gestión sanitaria, argumentando mayor eficiencia para proveer servicios mediante la colaboración entre sector público y privado. Este modelo utilizado, principalmente para infraestructura y servicios públicos, se promueve como una solución para problemas de financiación y gestión en contextos donde el Estado enfrenta recursos limitados. La expansión de las APP en América Latina comenzó en los años 1990 con las reformas neoliberales que favorecen la participación privada en la gestión pública. Sin embargo, son controversiales particularmente en salud, donde la privatización e inequidad en el acceso a servicios son tema de debate.

En este contexto, estudiar los mecanismos institucionales que han posibilitado la expansión de las APP en el Sistema General de Seguridad Social en Salud (SGSSS), da Colombia, permite comprender estos modelos de gestión sanitaria. Aunque algunas voces celebran su capacidad para mejorar la eficiencia y calidad, otras advierten que favorecen la concentración de recursos en manos privadas, a expensas de la equidad, del goce efectivo del derecho a la salud y del debilitamiento de la salud pública, generando múltiples disputas políticas que han sido documentadas en la literatura ^1^ ^,^ ^2^.

Esta investigación se preguntó por los dispositivos institucionales y normativos que han impulsado las APP, favoreciendo la concentración de recursos en actores privados y el debilitamiento del sector público. Además, examinó las transformaciones del sistema de salud a partir de la expansión de estas asociaciones, así como las tensiones que generan. Finalmente, evidencia las propuestas y disputas recientes sobre la orientación del sistema.

## Metodología

Se realizó un estudio de caso enfocado en los dispositivos legales e institucionales que sustentan el modelo de APP; fue descriptivo en los términos de Yin ^3^ e instrumental conforme Stake ^4^. Descriptivo en tanto se centra en la exploración y documentación del caso con el fin de comprender su complejidad, e instrumental en la medida en que el caso es una herramienta para comprender un fenómeno específico. Las fuentes primarias incluyeron leyes (10, periodo de 1993-2024), decretos (40, periodo de 1995-2022), resoluciones (66, periodo de 1995-2022), circulares (6, periodo de 1995-2022), sentencias de la Corte Constitucional (2, periodo de 2008 y 2014), y notas de prensa (342, periodo de 2009-2024). Fueron seleccionadas aquellas normas relacionadas con el aseguramiento y la prestación de servicios que regulan asuntos financieros, administrativos y funciones de actores públicos y privados. Adicionalmente, se realizó una revisión crítica de literatura especializada, para contextualizar y profundizar los debates sobre las APP. Para las fuentes primarias se realizó un análisis temático así: familiarización con los datos, generación de categorías iniciales, búsqueda, revisión, definición y denominación de temas ^5^, así: APP y sus adaptaciones para Colombia; transformaciones del SGSSS según las funciones de rectoría, financiamiento, articulación y prestación de servicios según el modelo de pluralismo estructurado ^6^ y disputas actuales del sistema de salud. Este abordaje permitió comprender los arreglos institucionales y algunas implicaciones de estas alianzas en términos de incidencia política, derecho a la salud y sostenibilidad del sector.

## Resultados y discusión

### El modelo de APP y sus adaptaciones en Colombia

Aunque el modelo de APP se promueve en América Latina desde los anõs 1990 en el contexto de reformas estructurales impulsadas por el llamado “Consenso de Washington”, su consolidación más significativa ocurrió en los años 2000. Estas asociaciones fueron inicialmente impulsadas argumentando mejorar la infraestructura pública y la provisión de servicios mediante la colaboración con el sector privado. Sin embargo, es más reciente la formalización de marcos normativos específicos en países como Chile, con la Ley de Concesiones de Obras Públicas de 2010, y México, con la Ley de Asociaciones Público-Privadas de 2012. En Colombia, aunque la *Constitución Política de 1991* abrió la puerta a la participación de los actores privados, fue con la *Ley 1.508* de 2012 que se expidió el régimen jurídico de las APP ^7^.

Este enfoque de gestión es respaldado por organismos financieros internacionales que las promueven como mecanismo eficiente para la provisión de bienes públicos. Esto enmarcado dentro del modelo de nueva gobernanza pública, entendida como “*el desarrollo integral, económico, social e institucional que no puede ser obra exclusiva del gobierno, dado que los recursos del Estado, aunque amplios, son limitados*” ^8^ (p. 4). Entonces, las APP son vistas como una estrategia para generar valor público. Sin embargo, en gobiernos con baja capacidad de regulación, estas alianzas pueden transformarse en mecanismos de cooptación de recursos públicos.

El Banco Interamericano de Desarrollo (BID) define estas alianzas como contratos a largo plazo entre una entidad pública y una privada ^9^, en los cuales esta última asume la responsabilidad de gestionar un activo o servicio público, bajo el argumento de ofrecer mayor calidad a menor costo y trasladar riesgos del sector público al privado. Bajo este esquema, el sector público debe concentrarse principalmente en la regulación y supervisión. En el ámbito de la salud, las APP han sido implementadas en proyectos como construcción de infraestructura hospitalaria, mercado de seguros, administración de recursos y prestación de servicios.

Sobre este modelo de gestión sanitaria hay balances, evaluaciones e informes de organismos internacionales que suelen mostrar los avances y aspectos a mejorar, pero poco cuestionan este modelo ^9^ ^,^ ^10^, asimismo, voces críticas como la European Network on Debt and Development (Eurodad) y la Red Latinoamericana por Justicia Económica y Social (Latindadd), instan a las instituciones financieras internacionales y a los gobiernos para detener la promoción de lo que llamaron la ideología de las APP. Dichos actores plantean que ese modelo amenaza con socavar los objetivos sociales en favor de beneficios particulares. Argumentan que, tales contratos no necesariamente contribuyen a optimizar la gestión de recursos públicos en la prestación de servicios de salud, considerando que pueden ser un negocio caro y riesgoso; no hay evidencia empírica sobre resultados positivos para su desarrollo; y pueden repercutir de forma negativa en el sistema de salud y su gobernanza ^11^.

Además, un análisis de países como Australia, España y Reino Unido muestra que la construcción de hospitales bajo este modelo ha sido más costosa que los métodos tradicionales ^12^. Sin embargo, también menciona aspectos positivos como el cumplimiento de tiempos y presupuestos, sin evidenciar que fueran de mejor calidad. Para organismos como la Comisión Económica para América Latina y el Caribe (CEPAL), esta forma de gestión es relevante en temas de infraestructura y en regiones como América Latina con bajo ingreso fiscal y alta deuda pública ^13^.

Los estudios que cuestionan los resultados y el impacto de estas asociaciones consideran que, mientras su popularidad crece, las evidencias sobre su efectividad son contradictorias. Diferentes autores ^14^ sugieren que, en lugar de confiar en el entusiasmo político, es fundamental realizar evaluaciones críticas, objetivas y no sesgadas por actores involucrados en la implementación. En esta misma línea, Laurell ^15^ señala que las APP en salud son una forma de privatización que incrementa los costos de operación para generar ganancias a las empresas privadas sin garantizar una atención adecuada.

En investigaciones sobre la implementación de las APP, sobresale una comparación entre Colombia, Chile e Inglaterra que muestra que en Colombia son costosas y poco efectivas, mientras que en Chile los servicios de salud prestados bajo esta modalidad han sido ineficientes en comparación con los servicios públicos ^16^. Otros autores reportan los riesgos de estos mecanismos en gobiernos afectados por altos índices de corrupción, como suele pasar en América Latina, donde este tipo de licitaciones y contratos están sometidos a intereses particulares y conducen a la apropiación de recursos públicos bajo el discurso de eficiencia y eficacia ^17^ y, desde 2010, se discute su efecto sobre el acceso a servicios y la equidad ^18^.

Además, se han analizado las implicaciones en la atención primaria, concluyendo que, aunque existen numerosos desafíos en la implementación, las APP pueden ser un mecanismo efectivo para aumentar el acceso de comunidades precarizadas, siempre y cuando se implementen, con marcos regulatorios sólidos y transparencia ^10^. Aunque no hay evidencias concluyentes, existen argumentos a favor y en contra de esta figura. Como señala Cunill ^19^, debido a la falta de evidencia sobre los efectos de las APP en América Latina, resulta fundamental profundizar tanto en el debate teórico y político como en los análisis sobre la interacción entre estas alianzas y el rol del Estado como garante de derechos.

En Colombia, el Consejo Nacional de Política Económica y Social (CONPES 3.615 de 2009) ^20^, definió las APP como “*un instrumento de vinculación de capital privado, que se materializa en un contrato entre una entidad estatal y una persona natural o jurídica de derecho privado para la provisión de bienes públicos, incorporando la retención y transferencia de riesgos entre las partes*” (*Ley 1.508* de 2012 ^7^). Esta regulación corresponde a distintos tipos de alianzas para el desarrollo de temas como infraestructura pública, servicios de educación, transporte y salud. Este último se ha caracterizado por la progresiva participación del sector privado, especialmente en la administración y prestación de servicios bajo la figura de las Entidades Promotoras de Salud (EPS), en su mayoría empresas privadas que se encargan de la gestión del aseguramiento, la intermediación financiera de los recursos entre el Estado y los prestadores de servicios, la gestión del riesgo en salud y la supervisión y el control del uso de servicios.

Este modelo de gestión en salud involucra temas más amplios relacionados con la gobernanza y las dinámicas de poder, en tanto comprende aspectos relacionados con la definición de reglas de juego formales e informales, roles de actores públicos y privados en la toma de decisiones, disputas por los recursos y tipos de arreglos institucionales que tienen efectos sobre cobertura, calidad y acceso a los servicios de salud ^21^ ^,^ ^22^.

### Transformaciones del SGSSS y mecanismos para fortalecer actores privados

Las transformaciones del SGSSS en los años 1990 generaron un conjunto de cambios regulatorios, institucionales y contractuales que posibilitaron el traslado de recursos públicos al sector privado, a través de APP, y en consonancia con lo que se ha denominado la financiarización de la economía ^23^. El análisis que se presenta en esta sección se organiza temáticamente siguiendo las funciones de los sistemas de salud planteadas en el modelo de pluralismo estructurado ^6^.

Las APP se legitimaron en el artículo 48 de la *Constituición Política de Colombia* que, aunque estableció la seguridad social como servicio público prestado bajo la “*dirección, coordinación y control del Estado*” con participación de particulares, abrió las puertas para la construcción de un mercado de servicios de salud materializado con la *Ley 100* de 1993 ^24^. Previamente, Colombia había intentado articular, a través del Sistema Nacional de Salud, diferentes subsectores: público, seguridad social y privado, sin embargo, tal aspiración no se concretó y constituyó una fractura del sistema de salud. En los años 1990, la crítica internacional y nacional a los sistemas de salud se profundizó con argumentos como baja cobertura de la seguridad social, ineficiencia en el manejo de recursos, burocratización, entre otros, presionando procesos de reforma que llevaron inicialmente a la descentralización en salud con la *Ley 10* de 1990 ^25^ y, posteriormente la creación del SGSSS (*Ley 100* de 1993 ^24^).

La primera transformación corresponde a la rectoría, caracterizada por una restricción creciente de la democracia en el proceso de toma de decisiones estratégicas. Inicialmente, el sistema organizó las competencias de dirección y regulación entre el Ministerio de Salud y Protección Social y el Consejo Nacional de Seguridad Social en Salud (CNSSS), conformado por 13 representantes: los ministros de Salud, Trabajo y Hacienda, un representante de EPS, uno de Instituciones Prestadoras de Servicios de Salud (IPS), dos de pequeñas y medianas empresas, uno de asociaciones de usuarios, uno de profesionales de la salud, uno de municipios y otro de departamentos. Los mecanismos básicos de regulación establecidos en el SGSSS son el Plan Obligatorio de Salud (POS), denominado Plan de Beneficios en Salud (PBS) desde la Ley Estatutaria de Salud ^26^, y la Unidad de Pago por Capitación (UPC), que buscaban regular el mercado sanitario y sus asimetrías a través del CNSSS ^27^.

El CNSSS fue eliminado luego de 14 años de existencia, por la *Ley 1.122* de 2007 ^28^ que en su lugar creó la Comisión de Regulación en Salud (CRES), transfiriéndole las funciones de dirección. La CRES estuvo adscripta al Ministerio de Salud y Protección Social, tuvo secretaría técnica y presupuesto propio. Estuvo conformada por siete miembros: los ministros de Hacienda y Protección Social, un representante de universidades, uno de centros de investigación en salud y otro de centros de investigación en economía, uno de asociaciones de usuarios y otro de profesionales de salud. Estos últimos cinco fueron considerados “expertos” de tiempo completo y elegidos por la Presidencia de la República; sus funciones se centraban en la definición del POS y la UPC ^28^.

La CRES, funcionó cinco años, realizando actividades de regulación, relacionadas con las órdenes de la Sentencia T-760/08 emitida por la Corte Constitucional ^29^, como la actualización del POS y la unificación por etapas de los planes de beneficios de los regímenes subsidiado y contributivo ^30^. Pese a tener rol de regulador, le fueron asignadas tareas técnicas, dejando como única función regulatoria el establecimiento del manual tarifario para la prestación de servicios de salud ^31^. En 2012, la CRES fue suprimida y sus funciones transferidas al Ministerio de Salud y Protección Social ^32^ ^,^ ^33^. Desde entonces no existe otro espacio colegiado de regulación y rectoría.

En suma, los actores vinculados a los procesos de dirección y regulación planteados en la *Ley 100* de 1993 fueron restringidos. El CNSSS tenía participación de actores gubernamentales, privados, trabajadores y usuarios, sin embargo, aunque estos últimos tenían voz y voto, la posibilidad de incidir en las decisiones era marginal dadas las mayorías gubernamentales ^34^. Posteriormente, la CRES tuvo participación y autonomía más restringidas, así como capacidad regulatoria limitada ^31^. Si bien en 2014 se creó la “Instancia de Coordinación y Asesoría del SGSSS” ^35^, los espacios de participación en las decisiones estratégicas fueron cada vez menores para actores subalternos y más favorables a privados.

La segunda transformación corresponde a la financiación. Previo a la reforma de los años 1990, el país invertía entre el 5,5% y el 6% del Producto Interno Bruto (PIB) en salud, del cual cerca del 50% se destinaba a la seguridad social que cubría menos del 20% de la población y el otro 50% era para las personas no cubiertas por la seguridad social ^36^. Del total del gasto en salud, el 55% correspondía a recursos públicos y el 45% al privado, para 2020, el gasto sanitario como proporción del PIB había subido a 7,8%, del cual el gasto público era 75% y el privado, 25% ^37^.

Financieramente, el SGSSS está dividido, por un lado, en los recursos del régimen contributivo que corresponden a las cotizaciones a la seguridad social de trabajadores y empleadores. La *Ley 100* de 1993 las fijó en 12% y posteriormente la *Ley 1.122* de 2007 ^28^ las incrementó a 12,5% (8,5% el empleador, previamente 8% y 4% el trabajador). Además, quienes ganan más de 4 salarios mínimos aportan el 1% al fondo de solidaridad. La transformación financiera más importante del regimene contributivo ocurrió con la *Ley 1.607* de 2012 ^38^, que disminuyó el aporte de empleadores sustituyéndolo por un Impuesto sobre la Renta para la Equidad, correspondiente al 8%, pagado por sociedades y personas jurídicas, del cual el 4,4% era para la salud. Este impuesto fue eliminado con la *Ley 1.819* de 2016 y se dispuso que un porcentaje del impuesto a la renta sería para la salud ^39^.

La financiación del régimen subsidiado proviene de varias fuentes: el porcentaje destinado a salud de los Ingresos Corrientes de la Nación que son transferidos a los municipios; recursos de situado fiscal transferidos a departamentos; recursos de la subcuenta de solidaridad del Fondo de Solidaridad y Garantía (FOSyGA); aportes de Cajas de Compensación Familiar; impuesto a la cerveza, juegos de azar y armas; y aportes adicionales de municipios y departamentos.

La regulación económica se ha hecho a través de la UPC, asimismo, se ha disminuido la brecha de financiación entre regímenes de afiliación ^40^. Adicionalmente, los recobros han sido el mecanismo por el cual las EPS cobran al Estado servicios de salud no incluidos en el POS, autorizados por comités o juntas técnicas u ordenados por el sistema judicial. A partir del 2008 hubo un incremento importante de los recobros (aproximadamente USD 260 millones). En 2010 se estimaron en alrededor de USD 546 millones y, entre 2010 y 2018, crecieron en un 31%, en 2019 llegaron aproximadamente a USD 970 millones ^37^ ^,^ ^41^. La mayor parte de los recobros están relacionados con medicamentos y tecnologías, muchas de ellas cubiertas por el sistema ^42^. Con la *Ley 1.955* de 2019 se establecieron “techos o presupuestos máximos”, intentando controlar el crecimiento de este gasto ^43^. En 2024, el giro total por UPC alcanzó USD 20.000 millones aproximadamente y el de techos máximos USD 907 millones, ambos representando incrementos respecto de 2023 ^44^.

En 2015, con la creación de la Administradora de los Recursos del Sistema General de Seguridad Social en Salud - ADRES (*Ley 1.753* de 2015) ^45^, se cristalizó el cambio más positivo y crítico para la función de financiamiento en salud. Esta nueva entidad pública de naturaleza especial, adscripta al Ministerio de Salud y Protección Social, busca mayor control sobre los recursos públicos ^46^, convirtiéndose en el gestor de los recursos a nivel nacional, especialmente para mejorar la oportunidad del pago a los prestadores de servicios ^44^.

La tercera transformación corresponde a la función de articulación, materializada a través de las aseguradoras en salud. La *Ley 100* de 1993 desarrolló un mercado de servicios de salud basado en aseguramiento individual, creando un nuevo actor para administrar los recursos de la atención y gestionar el riesgo en salud (EPS). Durante la implementación del sistema se produjo la concentración del aseguramiento en EPS privadas.

Inicialmente, para la organización del régimen subsidiado se permitió su administración a una variedad de nuevas y viejas instituciones: Empresas Solidarias de Salud (ESS), Cajas de Compensación Familiar y EPS creadas para tal fin ^47^. Asimismo, en 1994 se autorizó a las Entidades Territoriales para administrar el régime subsidiado con la creación de EPS públicas ^48^. Esta multiplicidad de instituciones y la dispersión geográfica de la población subsidiada generaron atomización de la afiliación y riesgos financieros. Las ESS fueron una novedad, pensadas como instituciones de la economía solidaria, creadas y gestionadas por comunidades, fueron espacios para la participación comunitaria en la administración y gestión de su salud a través del POS subsidiado ^49^. Muchas de estas organizaciones desaparecieron; en 1997 había 175 ESS ^50^ y para junio de 2024 quedan apenas dos EPS cuyo origen fueron ESS.

De otro lado, entre EPS y Administradoras del Régimen Subsidiado (ARS) en 1996, había 26 instituciones, 7 públicas y 19 privadas ^18^; entre las públicas se contaba el Instituto de Seguro Social (ISS), Cajanal, Caprecom, todas procedentes del antiguo sistema de salud. Las EPS y ARS públicas más grandes del país fueron liquidadas entre 2003 y 2015: Calisalud, Cajanal, ISS y Caprecom ^51^. Según el último informe de la Contraloría General de la República, desde la entrada en vigor de la reforma, se autorizó la creación de 157 EPS, de las cuales actualmente apenas 29 continúan funcionando - y solo seis cumplen los requisitos financieros - las demás fueron liquidadas o están en proceso, “*lo cual refleja la fragilidad del modelo*” ^44^ (p. 9).

Desde el inicio del actual sistema, estas instituciones han tenido la responsabilidad de afiliar a la población, manejar recursos, organizar y contratar una red de prestadores, garantizar la atención, auditar y pagar las cuentas. Durante los 31 años del SGSSS ha habido un gran debate sobre el rol de estos actores, con críticas alrededor de: múltiples barreras de acceso a los servicios de salud, altos costos de intermediación, integración vertical, falta de pagos oportunos a los prestadores con carteras morosas muy altas, cuestionamientos por corrupción, entre otros problemas. Las múltiples contiendas por la orientación del sistema de salud han puesto en el corazón del debate la pertinencia de las aseguradoras privadas, cuestionando este tipo de APP ^2^.

Este debate se da en medio de dos fenómenos adicionales: de un lado, la casi universalización de la cobertura en salud - que pasó de 29,21% en 1995 a 98,93% en 2023, para lo cual fue clave la Sentencia T-760/08 de la Corte Constitucional, que reconoció la salud como un derecho fundamental y ordenó al gobierno nacional, entre otros asuntos, la equiparación de los planes de beneficios entre los dos regímenes, asunto que era una promesa incumplida hasta entonces. De otro lado, actualmente hay mayor concentración, no solo de EPS privadas, sino de afiliados a ellas. A marzo de 2024 había 13,63 millones de personas afiliadas EPS públicas o mixtas y 35,84 millones a EPS privadas, adicionalmente, 2,23 millones de personas estaban en regímenes de excepción. En suma, a 31 años de la reforma, las ESS y las EPS públicas son minoritarias tanto en cantidad como en número de afiliados, y las EPS privadas se han fortalecido.

La cuarta transformación se ha enfocado en la prestación de servicios de salud. Entre los cambios ocurridos en la prestación de servicios resaltan tres aspectos que han llevado a la empresarización: la transformación de los antiguos hospitales públicos en Empresas Sociales del Estado (ESE); incremento de la proporción de prestadores privados frente a los públicos; y la exigibilidad jurídica del derecho a la salud. La transformación de los hospitales públicos implicó cambios jurídicos, económicos e institucionales al exigirles patrimonio propio y autonomía jurídica, administrativa y económica. Las ESE pasaron de recibir recursos de subsidio a la oferta a gestionar recursos para su funcionamiento, compitiendo en el mercado de salud con prestadores privados por los contratos.

Entre los efectos documentados están las crisis hospitalarias recurrentes. La primera crisis se registró entre 1998 y 2002, generando una importante movilización social ^2^ que persiste hasta la actualidad, conduciendo a cierres como el del emblemático Hospital Universitario San Juan de Dios en Bogotá, fundado en 1723 y cerrado en el 2001. Además, las ESE han sufrido múltiples procesos de reestructuración, bajo políticas gubernamentales como el CONPES 3204/2001-2002 y la política de prestación de servicios del 2005, orientadas especialmente hacia la flexibilización en las formas de contratación de trabajadores de la salud para reducir costos de producción ^52^. Pese a las reestructuraciones, la crisis ha persistido. Entre 2012 y 2019, el Ministerio de Salud y Protección Social emitió ocho resoluciones estableciendo criterios para definir un Índice de Riesgo de prestadores públicos, cuyo indicador central fue la solvencia financiera entendida como “*la capacidad de financiar las obligaciones operacionales corrientes y no corrientes de las instituciones, frente a los ingresos operacionales corrientes*” ^52^ (p. 4).

El crecimiento de las deudas de los aseguradores con los prestadores explica parte de dicha crisis, llevando a medidas de emergencia como el “Acuerdo de Punto Final”, propuesto por el expresidente Duque, para preservar la sostenibilidad financiera del sector ^43^. El Acuerdo consistía en la revisión, reconocimiento y pago de deudas causadas entre los diferentes actores del SGSSS; actualización del PBS; control de precios de medicamentos; negociación y compras centralizadas y la definición de valores máximos de recobro. La deuda de las 29 EPS, a 31 de diciembre de 2024 con las IPS, alcanzó COP 32,9 billones (aproximadamente USD 1.765 millones) ^44^.

**Figura 1:**
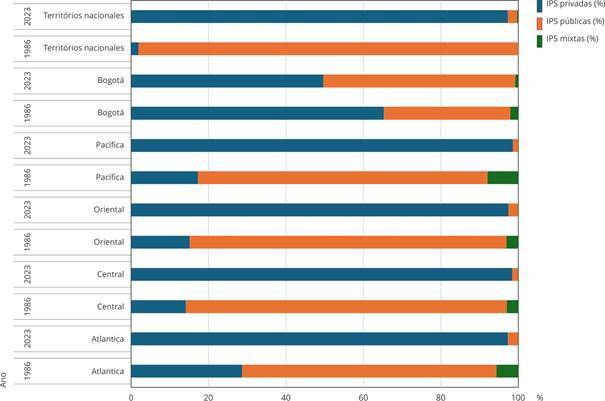
Proporción de Instituciones Prestadores de Servicios de Salud (IPS) por región según su naturaleza. Colombia, 1986 y 2023.

El crecimiento de los prestadores privados se evidencia al comparar que, hacia finales de los años ochenta, la mayoría de los servicios de salud en el país eran de carácter público - con excepción de Bogotá − mientras que para 2023 la mayor parte de las IPS eran privadas ^53^ (Figura 1).

Fuente: Uribe et al. ^53^.

En cuanto a la exigibilidad del derecho a la salud en el país, la población colombiana ha utilizado de manera sistemática la acción de tutela como una estrategia de exigibilidad jurídica, pese al incremento de la cobertura del aseguramiento y la equiparación de planes de beneficios. Ello es evidenciado por la instancia defensora de los derechos humanos en Colombia ^54^ (Tabla 1).

**Tabla 1:** Acción de tutela en salud. Colombia, 2000-2022.

Año	Tutelas	Afiliados	Proyección poblacional	Crecimiento de tutelas (%)	Tutelas por 10.000 afiliados	Tutelas por 10.000 habitantes
2000	24.843	23.017.680	40.295.563	16,6	10,8	6,2
2001	34.319	23.806.139	40.813.541	38,1	14,4	8,4
2002	42.734	24.194.854	41.328.824	24,5	17,7	10,3
2003	51.944	25.413.865	41.848.959	21,6	20,4	12,4
2004	72.033	30.040.650	42.368.489	38,7	24,0	17,0
2005	81.017	33.881.988	42.888.592	12,5	23,9	18,9
2006	96.226	36.461.003	43.405.956	18,8	26,4	22,2
2007	107.238	38.655.698	43.926.929	11,4	27,7	24,4
2008	142.957	39.561.521	44.451.147	33,3	36,1	32,2
2009	100.490	38.681.796	44.978.832	-29,7	26,0	22,3
2010	94.502	40.047.220	45.509.584	-6,0	23,6	20,8
2011	105.947	41.899.763	46.044.601	12,1	25,3	23,0
2012	114.313	42.854.862	46.581.823	7,9	26,7	24,5
2013	115.147	42.879.901	47.121.089	0,7	26,9	24,4
2014	117.746	43.515.870	47.661.787	2,3	27,1	24,7
2015	150.728	44.633.177	48.203.405	28,0	33,8	31,3
2016	164.274	46.404.041	48.747.708	9,0	35,4	33,7
2017	197.655	46.161.893	49.291.609	20,3	42,8	40,1
2018	207.734	46.623.525	49.834.240	5,1	44,6	41,7
2019	207.368	47.764.493	50.374.478	-0,2	43,4	41,2
2020	109.940	44.797.533	50.912.429	-46,9	24,5	21,6
2021	92.506	48.231.850	51.049.498	-36,9	19,2	18,1
2022	156.412	51.387.502	51.682.692	69,1	30,4	30,3

Fuente: Huerta Gutiérrez et al. ^54^.

### Disputas recientes por la reforma al sistema de salud (2020-2024)

En marzo de 2020, la declaración de emergencia sanitaria en Colombia por la pandemia de COVID-19 marcó una inflexión para el sistema. Como se ha visto, este venía enfrentando críticas por problemas estructurales ^55^. La pandemia, además de evidenciar debilidades acumuladas, detonó una demanda ciudadana para reformar el SGSSS.

En esta emergencia de salud pública, que representó el mayor desafío para los sistemas de salud, el papel del Estado fue central para garantizar oportunidad y calidad en la respuesta. El número de camas de Unidades de Cuidados Intensivos aumentó de 5,300 a 12,500 a finales de 2021 ^52^ ^,^ ^55^. Ello, gracias a iniciativas del gobierno nacional que buscaron reducir la fragmentación en la prestación de servicios y coordinar la respuesta mediante entidades clave como el Ministerio de Salud, el Instituto Nacional de Salud, la Superintendencia Nacional de Salud y la ADRES. La pandemia reabrió el debate sobre el papel del Estado frente a la participación privada en el sistema de salud.

En 2022, con la llegada del presidente Gustavo Petro, se intensificaron las presiones para reformar el sistema. Petro, crítico de la intermediación financiera de las EPS, propuso una reforma estructural que buscaba eliminar esta intermediación, fortalecer la atención primaria y devolver al Estado el control directo sobre los recursos del sistema. La designación de Carolina Corcho como ministra de salud, reconocida por su papel en movimientos sociales y gremios médicos, marcó el inicio de un intento ambicioso de transformación. Sin embargo, su propuesta encontró una fuerte resistencia de actores privados de la salud y gremios económicos, quienes denunciaron la falta de diálogo y advirtieron supuestos riesgos para la libertad de elección de los usuarios. Los partidos políticos tradicionales, defensores del mercado en salud, también se opusieron ^53^.

El proyecto de reforma de 2022 buscaba transformar las EPS en “gestoras de salud y vida”, dejando en manos del Estado la administración de los recursos financieros sin haber llegado a acuerdos. Para finales de 2023, continuaron las negociaciones y concesiones sin lograr avances en la transformación del sistema. En 2024, un nuevo intento de reforma presentó un enfoque más conciliador, que incluía la participación de las EPS en un modelo con enfoque en atención primaria, pero también limitaba su capacidad de intermediación financiera. A pesar de estos esfuerzos, este proyecto también fue archivado, debido a conflictos de interés entre senadores y la influencia de intereses privados (Cuadro 1). Se espera que durante el último periodo legislativo del gobierno Petro (julio 2025 a agosto 2026) se retome la discusión de un nuevo proyecto de ley, que se gesta en medio de viejas tensiones entre intereses privados y públicos relacionados con el papel de los aseguradores y el manejo de los recursos del sistema.

**Cuadro 1:** Proyectos de Ley para reformar el sistema de salud. Colombia, 2020-2024.

AÑO	NÚMERO DE PROYECTO DE LEY	PROPUESTA GENERAL	DEBATES	PUNTOS PRINCIPALES	FECHA DE ARCHIVO
2020	*Proyecto 010* de agosto 2020, Senado	Reforma al sistema de salud para fortalecer la atención primaria y la red pública	2 debates	Fortalecimiento de la atención primaria Mayor inversión en la red pública de hospitales Mejora en la contratación de personal médico	Archivado en **diciembre de 2020**
2021	*Proyecto 230* de marzo 2021, Cámara	Creación de un sistema único y público de salud	3 debates	Eliminación progresiva de las EPS Creación de un sistema público centralizado Garantía de acceso universal y gratuito	Archivado en **noviembre de 2021**
2021	*Proyecto 350* de julio 2021, Senado	Reforma para mejorar la eficiencia y transparencia en la administración de recursos del sistema de salud	2 debates	Auditorías obligatorias a las EPS Reducción de intermediarios Mayor control estatal	Archivado en **marzo de 2022**
2022	*Proyecto 120* de febrero 2022 Cámara	Propuesta para eliminar las EPS y crear un modelo de salud estatal	4 debates	Eliminación total de las EPS Creación de un modelo estatal de salud. Mayor inversión en infraestructura hospitalaria	Archivado en **agosto de 2022**
2022	*Proyecto 450* de septiembre 2022, Senado	Reforma para garantizar el acceso universal a medicamentos y servicios de salud	3 debates	Acceso gratuito a medicamentos esenciales Regulación de precios de medicamentos Ampliación de cobertura en zonas rurales	Archivado en **mayo de 2023**
2023	*Proyecto 200* de abril 2023, Cámara	Iniciativa para fortalecer la atención en zonas rurales y marginadas	2 debates	Creación de brigadas médicas móviles Inversión en infraestructura rural Programas de prevención y promoción de la salud en zonas apartadas Telemedicina como servicio básico Plataformas digitales para agilizar citas y trámites	En trámite (no archivado)
2023	*Proyecto 339* de septiembre 2023, Cámara	Reforma para garantizar la sostenibilidad financiera del sistema de salud y mejorar la calidad en la prestación de servicios	2 debates	Reestructuración del modelo de financiación de las EPS Fortalecimiento de la supervisión estatal Mejora en los mecanismos de atención al usuario	Archivado en **abril de 2024**
2024	*Proyecto 312* de febrero 2024, Senado	Reforma estructural al sistema de salud, con enfoque en la eliminación de las EPS y fortalecimiento de la red pública	1 debate (en curso)	Eliminación de las EPS y creación de un sistema público unificado Fortalecimiento de la red hospitalaria pública Mayor control estatal sobre los recursos y la prestación de servicios de salud	En trámite (no archivado)

EPS: entidades promotoras de salud.

Nota: archivo del proyecto refiere a que este no continúa su trámite legislativo debido a falta de consenso político.

Fuentes: elaboración propia con base en Huerta Gutiérrez et al. ^54^ y consultas en portales de internet del Congreso de la República.

## Reflexiones finales

El caso colombiano es un claro ejemplo de los riesgos de permitir el crecimiento sin límites de los actores privados en el sector salud. La correlación de fuerzas de estos actores ha acabado inclinando las decisiones a su favor. Además, el proceso de afianzamiento de las APP como mecanismo de gestión en salud no ha sido espontáneo, son 31 años de construcción de un marco institucional y normativo que las fortaleció. Las diversas disputas muestran que la consolidación de las APP ha fortalecido la lógica empresarial en el sistema de salud. Las modificaciones legislativas y administrativas han reforzado la posición de actores privados, quienes han logrado legitimar este modelo ante la ciudadanía. Aunque las APP han contribuido a expandir la infraestructura y la cobertura, su implementación ha fragmentado la prestación de servicios de salud y debilitado el papel del sector público en el manejo de los recursos. Si bien es fundamental mantener una red de prestadores público-privados, es necesario establecer el acceso efectivo y control estatal y ciudadano adecuado para priorizar el derecho a la salud sobre las dinámicas de rentabilidad. 

A pesar de la abundante literatura sobre el caso colombiano, este artículo aporta evidencia sobre los dispositivos institucionales y legales de las APP que han sido decisivos para consolidar al sector privado como actor dominante en el sistema de salud, no solo por la concentración de recursos que administran, sino por su poder creciente para orientar las decisiones de política pública en este ámbito.

Como limitaciones, dada la complejidad del caso colombiano, no fue posible describir ni profundizar en otras temáticas clave como los procesos de contratación entre actores y sus transformaciones en el tiempo, las condiciones laborales del personal de salud, ni la organización y gestión de las redes de prestación de servicios de salud, incluidas las tecnologías sanitarias, entre outros.

## Data Availability

Las fuentes de información utilizadas en el estudio se indican en el cuerpo del artículo.
